# A critically prolonged avalanche burial with recorded cardiac electrical activity and complete recovery - a case report

**DOI:** 10.1186/s13049-024-01230-0

**Published:** 2024-07-03

**Authors:** C. Bracco, G. Strapazzon, A. Sciolla, A. Dupuis, G. Lauria, L. Fenoglio

**Affiliations:** 1 Department of Internal Medicine, Santa Croce E Carle Hospital, Cuneo, Italy; 2 Institute of Mountain Emergency Medicine, EURAC Research, Bolzano, Italy; 3 Department of Emergency Medicine, Santa Croce E Carle Hospital, Cuneo, Italy

**Keywords:** Avalanche, Severe hypotermia, Hearth electrical activity

## Abstract

The probability of survival in avalanche accidents is time-dependent. Critically buried victims who undergo a long burial duration (over 60 min) face a possible mortality rate of over 80%. Understanding the physiological response during critical avalanche burial is crucial for improving rescue strategies and outcomes. We present the case of a 55-year-old male skier buried under an avalanche for 4 h and 51 min in the Italian Alps. Continuous heart rate monitoring revealed distinct phases of cardiac activity during burial. Despite severe hypothermia, the victim survived without extracorporeal rewarming. This case highlights the importance of continuous monitoring and appropriate on-site management in avalanche accidents. Factors such as the presence of an air pocket may positively influence survival. This case underscores the importance of comprehensive resuscitative measures and guidelines for managing avalanche victims with prolonged burial durations.

## Background

In Italy, an average of 100 avalanches occur every year, and the overall mortality rate of avalanche accidents is 25% [1, 2]. Survival probability in the absence of severe traumatic injuries is time dependent, and critically buried victims who undergo a long burial duration (over 60 min) face a possible mortality rate of over 80% [3]. Victims can survive if they can breathe under the snow and maintain sufficient delivery of oxygen and removal of carbon dioxide until they cool down [4]. The current resuscitation guidelines recommend transporting a victim buried for over 60 min with cardiovascular instability or in cardiac arrest but with patent airway to an Extracorporeal Life Support (ECLS) center [5, 6].

The pathophysiology of critical prolonged avalanche burial, especially with respect to hypothermia and circulatory dysfunction, remains largely unknown. Prospective data in the literature, especially on prolonged burial of over 60 min, are mainly derived from animal experiments. In 2012, Strapazzon et al. described the first recorded cardiac activity during prolonged avalanche burial, and confirmed potential long-term cardiac electrical activity in an avalanche victim with a patent airway. Unfortunately, the victim was unconscious and had no vital signs at extrication and did not survive [7]. In 2013 Heshl described an other case of completely 290 min burial with recorded cardiac activity but the victim had obstructed airway at extrication and vital signs were absent; asystole was confirmed with an electrocardiogram (ECG), and the epitympanic temperature was 24.0 °C. In accordance with current guidelines, the man was declared dead on site [8].

Here we describe the first case of a critical long burial of an avalanche victim with a continuous heart rate. Data were recorded during the burial and the outcome of the victim was favorable. This was also one of the few avalanche cases of a victim with severe hypothermia who was rewarmed successfully without ECLS techniques.

## Case presentation

A 55-year-old male skier was caught by an avalanche in Valle Grana, Piemonte, Italy. The accident occurred during the skier's descent at an elevation of 2,240 m above sea level. He was conscious at the time of avalanche hitting and kept thinking about how to get out of it and communicate with his wife. During this time he tried to keep his hands in front of his face to create room for breathing. In his memory he lost consciousness a few minutes after these thoughts.

He was located using an avalanche transceiver device, probed, and extricated by a team from the local Helicopter Emergency Medical Services (HEMS) after 4 h and 51 min of burial as he was alone and the call to the emergency services was delayed.

**Fig. 1:**
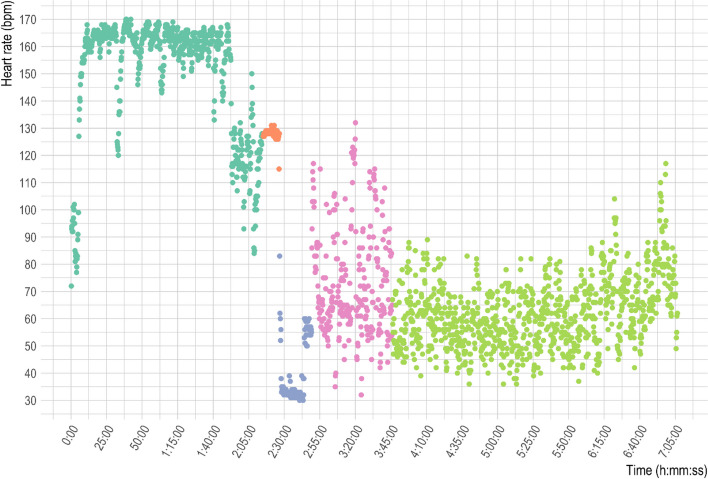
First green points: heart rate during ascent and resting on the top; orange points: first 11 min of burial; blue points: from 12 to 23 min: shift to bradycardia; pink points: 24 to 80 min: higher heart rate with a highly variable frequency; last green points: 80 to 291 min higher heart rate with a lower variability

Heart rate data were recorded during the ascent, descent, and the entire burial period by an Onmove 500 model watch (Decathlon, Villeneuve-d'Ascq, France) equipped with an optical sensor using the pulse photoplethysmography (PPG) system to detect heart rate at the wrist. Heart rate data before and during the burial are presented in Fig. 1. Before the burial, the heart rate (mean, 151 ± 22 bpm; range, 72–170 bpm) showed values that reflected physical effort. Over the course of the burial, four distinct phases of cardiac activity were apparent: (i) 1 to 11 min: sustained tachycardia with a mean of 128 ± 2 bpm (range 115–131 bpm); (ii) 12 to 23 min: shift to bradycardia with a mean of 40 ± 11 bpm (range 30–83 bpm); (iii) 24- 80 min: higher heart rate with a highly variable frequency and a mean of 75 ± 20 bpm (range, 32–132 bpm); and (iv) 80 to 291 min: heart rate with a lower variability and a mean of 63 ± 13 bpm (range, 36–117 bpm).

The victim was found in a supine position, with his head buried at a depth of one meter, but with a patent airway and a visible air pocket in front of the mouth and nose. Upon initial evaluation, he was unconscious (U) but he was breathing spontaneously (respiratory rate 20/min as reported in the emergency medical service form) and a central pulse was present. After ECG monitoring by the HEMS service, he was lifted on a stretcher (Kit Everest Carbon, Kong Italy) and air transported to the Cuneo Regional Hospital to provide ECLS. Warm fluids and fentanyl 50 + 50 mcg were administered during the helicopter transport via intraosseous access.

**Fig. 2:**
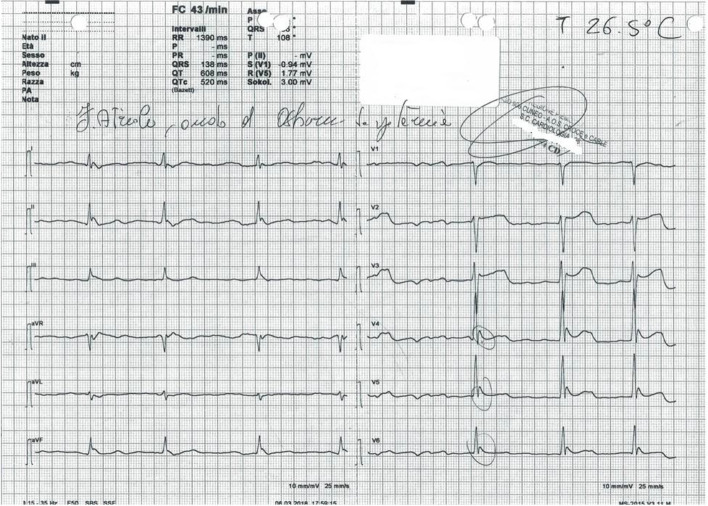
ECG at the arrival in Emergency Department showing a Osborn wave

**Fig. 3:**
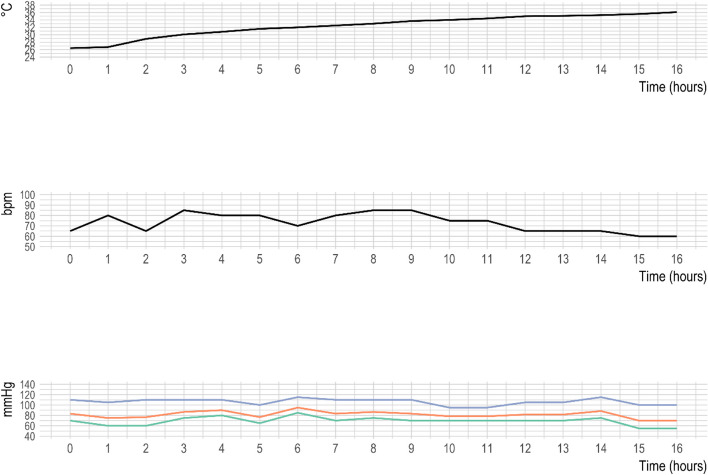
Esophageal core temperature, heart rate and blood pressure during recovery in the Intensive Care Department

Upon arrival at the Emergency Department, the patient was intubated after sedation (propofol and rocuronium) because of severe agitation; he had a heart rate of 48 bpm and the electrocardiogram documented atrial fibrillation, with a QRS duration of 136 ms, QTc interval of 520 ms, and an Osborne J wave (Fig. 2). Blood pressure was 125/75 mmHg, the Glasgow Coma Scale score was 9 (E4 V1 M4) and the esophageal core temperature was 26.4°C. Arterial blood gas analysis, with FiO2 at 100%, showed pH 6.98 (normal range 7.35–7.45), arterial oxygen pressure 169 mmHg (normal range 83–108), arterial carbon dioxide pressure 66.8 mmHg (normal range 35–48), bicarbonate 16 mmol/L, sodium 140 mEq/L (normal range 136–146), potassium 5.3 mEq/L (normal range 3.4–4.5), and lactate 11.6 mmol/L (normal range 0.3–1.2). Chest X-ray showed a small right pulmonary contusion, while head computed tomography and abdominal ultrasound were unremarkable. The victim was transferred to the Intensive Care Department, internal rewarming with warm crystalloids and active external rewarming using the 3M Bair Hugger system (3MTM, St Paul, MN) at a rate of 0.5°C per hour (Fig. 3) were continued; he was sedated and relaxed with cisatracurium (6 mg per hour) and propofol (20 mg per hour). The patient remained hemodynamically stable, without the need for vasopressors or other active internal rewarming systems. On the second day of hospitalization, the treatment was discontinued, and sedation was gradually reduced. After 17 h and 41 min from hospital admission, the victim's temperature reached 36.1°C, and active external rewarming was stopped. Sedation was suspended, and the patient was extubated.

**Fig. 4:**
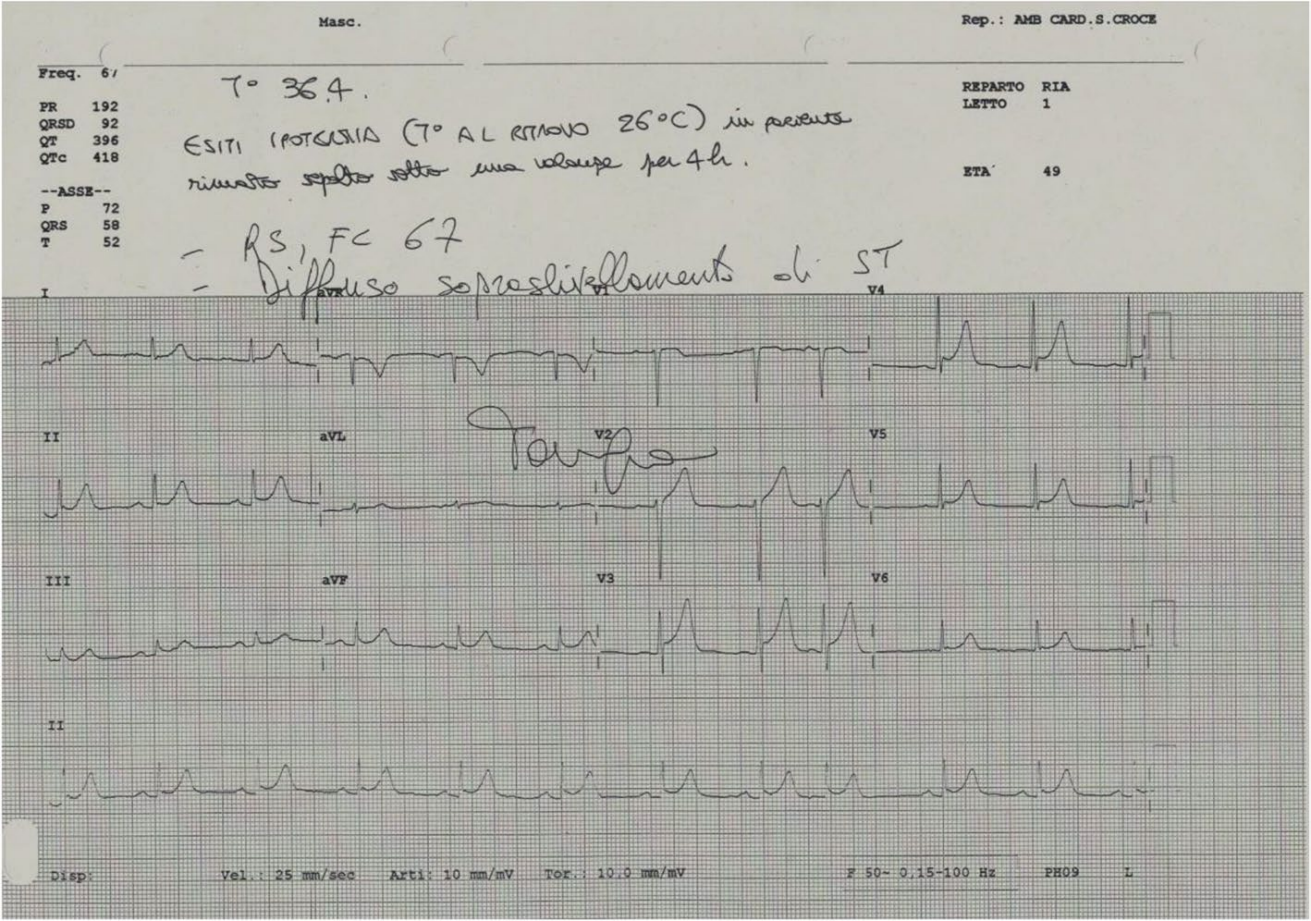
ECG at day 2 with normal core temperature

At this moment an ECG recorded in Intensive Care Unit showed a sinusal rhythm (Fig. 4).

On the third day of hospitalization, the victim was transferred to the Emergency Medicine ward, where he received intravenous hydration to address a creatine kinase level of 1230 U/L, ultimately reducing it to 302 U/L.

On the fourth day the patient was discharged from the hospital with a Cerebral Performance Score (CPC) score of one and in good overall condition. He resumed his job and continued his daily life and sports activity without any impairment.

## Discussion and conclusions

This case of survival with complete recovery following critical avalanche burial lasting 4 h and 51 min with severe hypothermia and maintained spontaneous circulation represents one of the longest durations ever recorded. Two aspects of the case are particularly remarkable. The first is that the heart-activity data on avalanche-burial in a human was recorded, and the second concerns the detailed management of a severely hypothermic victim after critical burial > 60 min without ECLS rewarming and no long-term complications.

No data are available on continuous heart-rate monitoring during critical avalanche burial > 60 min with a favorable outcome in humans. In our case, there was a high variability of the recorded heart rate for up to 80 min after the critical burial where three heart rate patterns were highlighted with an initial tachycardia, followed by bradycardia and a third phase with a higher heart rate and a highly variable frequency. After a phase of tachycardia in mild hypothermia, bradycardia is a well-known effect of cooling however it is not always observed during moderate accidental hypothermia [9, 10]. A sympathetically mediated increase subsequent to initial cooling, could contribute to large variations in heart rate. Our case showed a highly variable frequency in heart rate within 24 and 80 min of critical burial, which persisted, albeit at a lower frequency, after 80 min where a higher degree of hypothermia is expected.

The victim showed atrial fibrillation after he had been extricated. Atrial fibrillation is reported to have a high incidence in accidental hypothermia with a variable threshold of temperature. In humans with accidental hypothermia, ventricular fibrillation or asystole is also common in patients admitted with low core temperatures as cardiac repolarization is prolonged with an approximately linear relationship with decreasing core temperature [9]. Appropriate on-site management, gentle extrication from the snow and continuous monitoring [5, 7] probably prevented the occurrence of ventricular fibrillation/asystole and cardiovascular instability during the management of the victim.

Factors positively influencing the survival of critically buried avalanche victims [4, 11] were evident during the victim's extrication. These included the patency of the airway as well as a large air pocket which probably enabled the sufficient exchange of respiratory gases before cooling, as shown by the arterial blood gas analysis in the Emergency Department. The victim showed similar arterial blood gas analysis values to tests carried out on animals where low arterial oxygen pressure and high arterial carbon dioxide pressure complicate the pathophysiology of accidental hypothermia [12, 13]. Serum potassium and acidosis did not rise substantially and could be explained by a cooling before the critical level of hypoxia; in fact the victim had a core temperature after extrication of 26.4 °C and a positive outcome. Surprisingly similar data were reported in another case report of a victim buried for 127 min [14]. The patient’s cooling rate averaged 2.2°C per hour, which aligns with literature data indicating a range of 0.9–9°C per hour, and an average cooling rate of 3°C per hour [15, 16]. However, the transport of the victim to an ECLS center is of paramount importance in cases of incoming cardiac arrest or cardiovascular instability, as well as other possible complications as described during the rewarming of another long burial avalanche victim [14] and suggested by recent guidelines on accidental hypothermia and avalanche burial [5, 6].

HEMS providers should be prepared to manage avalanche victims who have been subjected to a long burial. This case confirms the potential long-term mechanical and cardiac electrical activity in an avalanche victim with a patent airway. The case emphasizes the importance of evaluating vital signs or ECG monitoring (in victims without signs of life) at extrication, ideally before moving the victim [5]. The case also suggests that avalanche victims extricated after more than 60 min of burial with a patent airway should receive full resuscitative measures, especially if they have an air pocket and no fatal traumatic injuries.

## Data Availability

All data generated or analysed during this study are included in this published article.
